# Genetic assessment reveals inbreeding, possible hybridization, and low levels of genetic structure in a declining goose population

**DOI:** 10.1002/ece3.8547

**Published:** 2022-01-24

**Authors:** Johanna Honka, Serena Baini, Jeremy B. Searle, Laura Kvist, Jouni Aspi

**Affiliations:** ^1^ Ecology and Genetics Research Unit University of Oulu Oulu Finland; ^2^ Department of Biology University of Rome “Tor Vergata” Rome Italy; ^3^ Department of Ecology and Evolutionary Biology Cornell University Ithaca New York USA

**Keywords:** *Anser fabalis*, citizen science, control region, hybridization, microsatellites, mitochondrial DNA, non‐invasive sampling

## Abstract

The population numbers of taiga bean goose (*Anser fabalis fabalis*) have halved during recent decades. Since this subspecies is hunted throughout most of its range, the decline is of management concern. Knowledge of the genetic population structure and diversity is important for guiding management and conservation efforts. Genetically unique subpopulations might be hunted to extinction if not managed separately, and any inbreeding depression or lack of genetic diversity may affect the ability to adapt to changing environments and increase extinction risk. We used microsatellite and mitochondrial DNA markers to study the genetic population structure and diversity among taiga bean geese breeding within the Central flyway management unit using non‐invasively collected feathers. We found some genetic structuring with the maternally inherited mitochondrial DNA between four geographic regions (*ɸ*_ST_ = 0.11–0.20) but none with the nuclear microsatellite markers (all pairwise *F*_ST_‐values = 0.002–0.005). These results could be explained by female natal philopatry and male‐biased dispersal, which completely homogenizes the nuclear genome. Therefore, the population could be managed as a single unit. Genetic diversity was still at a moderate level (average *H*_E_ = 0.69) and there were no signs of past population size reductions, although significantly positive inbreeding coefficients in all sampling sites (*F*_IS_ = 0.05–0.10) and high relatedness values (*r* = 0.60–0.86) between some individuals could indicate inbreeding. In addition, there was evidence of either incomplete lineage sorting or introgression from the pink‐footed goose (*Anser brachyrhynchus*). The current population is not under threat by genetic impoverishment but monitoring in the future is desirable.

## Graphical abstract

We studied large‐scale genetic population structure of declining taiga bean geese (*Anser fabalis fabalis*) in their breeding habitat using a citizen‐science approach. Over 2000 non‐invasively collected feathers were genotyped with microsatellite markers, and mitochondrial DNA sequences were analyzed in the 491 individual geese identified. We found very limited population structure, signs of inbreeding, and surprisingly, indications of hybridization with another goose species, the pink‐footed goose (*Anser brachyrhynchus*).

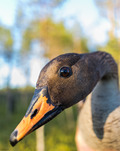

## 1 INTRODUCTION

**FIGURE 1:**
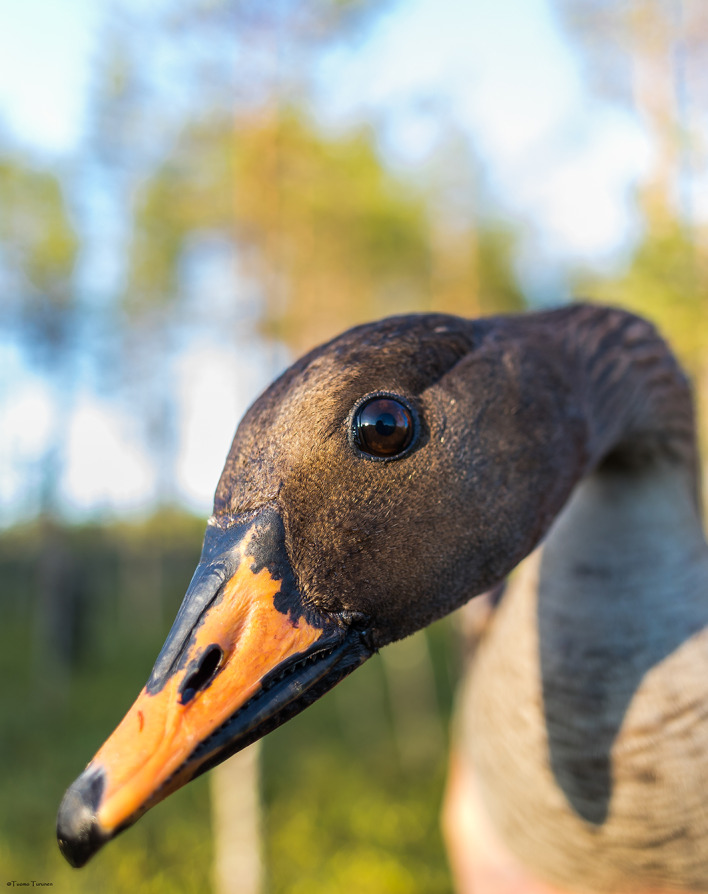
Taiga bean goose (*Anser fabalis fabalis*) sampled for this study during ringing. Photo: Tuomo Turunen

Knowledge of population genetic structure is essential for guiding management and conservation of species. The presence of highly divergent subpopulations with low levels of gene flow could warrant a status of separate management units (MUs; Palsbøll et al., 2007). Loss of genetic diversity and inbreeding depression, processes that especially affect small and fragmented populations, may lead to a loss of evolutionary potential and contribute to a higher extinction risk (Frankham, 2005). Harvesting can also lead to adverse genetic changes such as alteration of population subdivision (extirpation of local populations), loss of genetic variation, and selective genetic changes (reduction in certain phenotypes targeted by hunting) that may further compromise population viability (Allendorf et al., 2008). Thus, knowledge of genetic structure and diversity is especially important for managing harvested species and subspecies to ensure sustainable hunting. This has been our motivation to study the taiga bean goose (*Anser fabalis*; Figure 1), which is hunted over most of its range but has suffered a marked decline during recent decades (Fox et al., 2010). However, very little is currently known about the genetic population structure and diversity of the taiga bean geese in their breeding area.

**FIGURE 2:**
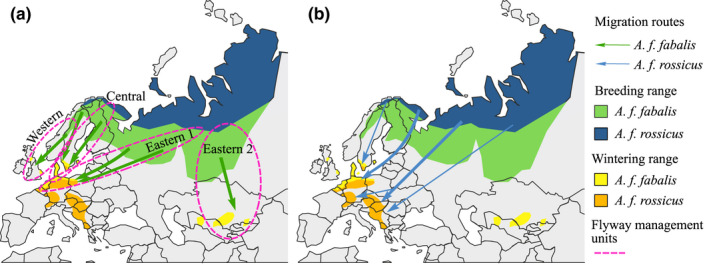
Breeding and wintering distributions of the taiga (*Anser fabalis fabalis*) and tundra (*Anser fabalis rossicus*) bean goose shown with different colors, and the autumn migration routes of (a) the taiga bean goose shown with green arrows and (b) the tundra bean goose shown with blue arrows. The flyway management units used in the International Single Species Action Plan (Marjakangas et al., 2015) for the taiga bean goose are also shown with dashed ellipses. Maps redrawn from BirdLife International (2018) and Marjakangas et al. (2015)

Most Holarctic goose populations are on the increase (Fox & Leafloor, 2018) to the extent that this is causing conflicts with humans, especially in agriculture (Fox & Madsen, 2017). However, a few goose populations are declining, one of them being the taiga bean goose (Fox et al., 2010; Fox & Leafloor, 2018) breeding in Northern Europe and Asia (Figure 2). The population size of the taiga bean goose has nearly halved from the 90,000–100,000 individuals in the 1990s (Nilsson et al., 1999) to the current estimate of 52,000 individuals for the total wintering population size (Fox & Leafloor, 2018). Although the exact cause of decline is uncertain, overharvesting is one plausible explanation. Since the taiga bean goose population is still open to hunting, sustainable management of the population is of crucial importance. Within this framework, an International Single Species Action Plan (ISSAP) was developed by The African‐Eurasian Migratory Waterbird Agreement (AEWA) to conserve the taiga bean goose (Marjakangas et al., 2015). Hunting of the Northeast/Northwest European population of the taiga bean goose can still be continued within the limits of agreed sustainable use within the ISSAP framework (Marjakangas et al., 2015).

The ISSAP for the taiga bean goose recognizes four subpopulations or flyway management units: Western, Central, Eastern 1, and Eastern 2 subpopulations (Marjakangas et al., 2015; Figure 2). These flyway units are distinguished by isotopic composition of feathers collected from bean geese from Western, Central, and Eastern 1 flyway units (Fox et al., 2017) but the units have not been confirmed genetically and it is not known if these units should be further subdivided into smaller management units. For effective management of the taiga bean goose, population genetic structure should be assessed in order to preserve genetic diversity. Previously, the population genetic structure of the taiga bean goose has only been studied within a limited area in Central Scandinavia belonging to the Western flyway management unit (de Jong et al., 2019).

The taiga bean geese are elusive, especially during the breeding period, and observing even neck‐banded geese in breeding areas is rarely possible due to the long escape distance of geese (Pirkola & Kalinainen, 1984a)—although camera traps have been shown to be a viable alternative for taiga bean goose monitoring on breeding grounds (Nykänen et al., 2021). Thus, catching geese and sampling blood would be very difficult and stressful for the birds. Developments in non‐invasive genetic assessments have allowed sampling of elusive and endangered species without the need to handle or even observe the animals (Taberlet et al., 1999). For birds, nest material and molted feathers provide a valuable source of DNA (Pearce et al., 1997; Segelbacher, 2002). The taiga bean geese perform a molt from the middle of June to the middle of August, and during this flightless period, they spend time in the wettest part of mires or in the vicinity of ponds, where they leave abundant cues of their presence such as tracks, signs of grazing, feces, and molted feathers (Pirkola & Kalinainen, 1984b). In our study, we utilized this source of non‐invasive feather samples collected by volunteers (citizen science) to study the genetic population structure of the breeding taiga bean geese.

Our aims were (1) to evaluate the utility of a citizen‐science approach in collecting genetic samples for the elusive taiga bean goose, (2) to assess the broad‐scale genetic structure among breeding taiga bean geese in Finland (consisting of a large part of the Central management unit), (3) to estimate genetic diversity within this population, (4) to evaluate the current effective population size and demographic fluctuations, and (5) to investigate if taiga bean geese hybridize with another subspecies of bean goose breeding in Europe, the tundra bean goose (*Anser fabalis rossicus*), and with the most closely related species, the pink‐footed goose (*Anser brachyrhynchus*). For this, we used highly variable microsatellites first to identify individuals and parentage and then to study variation at the population level. In addition, we defined the genetic structure of maternal lineages by sequencing the most variable part of the mitochondrial control region. This region was also used to verify the subspecies (see Honka et al., 2017).

## 2 MATERIAL AND METHODS

### 2.1 Study species

The taxonomy of the bean goose and the related pink‐footed goose (*A. brachyrhynchus*) has been controversial (Ruokonen & Aarvak, 2011). Currently, the bean goose is either split into two species (*A. fabalis* and *Anser serrirostris*; Sangster & Oreel, 1996) or treated as one species (*A. fabalis*) with four subspecies (*Anser fabalis fabalis*, *A. f. rossicus*, *Anser fabalis middendorffii*, and *Anser fabalis serrirostris*; Mooij & Zöckler, 1999). Based on a recent study using genome‐wide data, *A. f. fabalis* and *A. f. rossicus* should be classified as subspecies (Ottenburghs et al., 2020) and thus we follow here the classification of one species with four subspecies. From here on, we focus on the western subspecies and refer to *A. f. fabalis* as the taiga bean goose and *A. f. rossicus* as the tundra bean goose.

**FIGURE 3:**
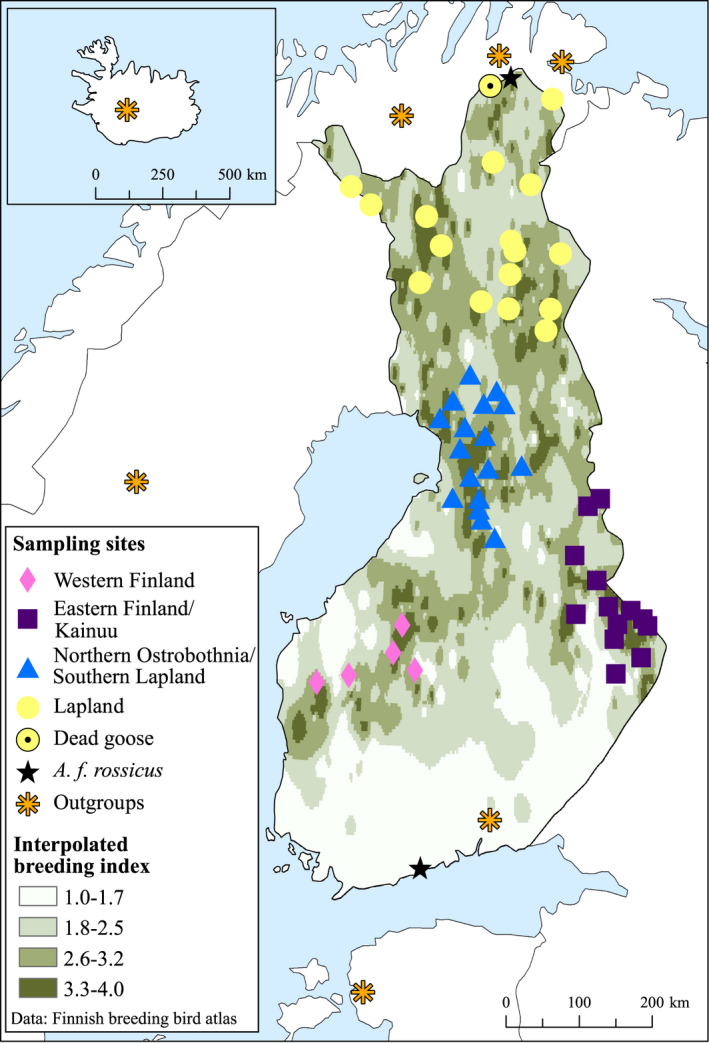
The breeding distribution of the taiga bean goose (*Anser fabalis fabalis*) in Finland and the approximate location of the sampling sites of the current study. The exact sampling sites are not shown in order to protect the breeding locations and the sites closer than 16 km were merged. Samples are divided here into four geographical regions shown with different symbols and colors. Samples of tundra bean goose (*Anser fabalis rossicus*) mtDNA are indicated with a star and one dead goose that was not ascertained as breeding is indicated with a black dot. The sampling sites of outgroups are also shown. The background breeding distribution map was created by interpolating the breeding category indices (1 = unlikely breeding, 2 = possible breeding, 3 = probable breeding, and 4 = confirmed breeding) of the Finnish Breeding Bird Atlas using the Inverse Distance Weighted method in ArcGIS software. The breeding index data are from: Results of the 3rd Finnish bird atlas. Finnish Museum of Natural History, University of Helsinki Luomus. Used with Creative Commons Attribution 4.0 license

The taiga bean goose breeding distribution covers the forested taiga area from Scandinavia to Western Siberia (Scott & Rose, 1996; Figure 2) and in Finland the “aapa” mire zone (see Laitinen et al., 2007) with core breeding area extending from Lapland to Northern Ostrobothnia (Pirkola & Kalinainen, 1984b; Figure 3). In Finland, the taiga bean goose is listed as vulnerable (the Red List of Finnish species; Lehikoinen et al., 2019) with the breeding population size estimated to be 1700–2500 pairs (i.e., 3400–5000 breeding individuals; Valkama et al., 2011). Subadults and failed breeders perform a molt migration to Novaya Zemlya (Nilsson et al., 2010; Piironen et al., 2021) and thus are not counted. In addition to the taiga bean goose, a very small number of tundra bean geese may breed in Finland in the most northernmost Lapland adjacent to their breeding range in Norwegian Finnmark (Aarvak & Øien, 2009; Figure 2). This subspecies is listed as near threatened in Finland (The Red List of Finnish species; Lehikoinen et al., 2019).

### 2.2 Sampling and DNA extraction

Bean goose feathers were collected from nests or brood‐rearing/molting sites in Finland or close to the Finnish border (Sør‐Varanger municipality, Norway) during the years 2006–2014 (*n* = 14) and 2016–2018 (*n* = 2127) or from taiga bean geese handled for ringing (capturing and marking of birds was done by the approval of Finnish Wildlife Agency License Number 2019‐5‐600‐01158‐8) in the years 2017–2018 (*n* = 20) (Figure 3). For geese that have bred successfully, the nest site is most probably close to the brood‐rearing/molting site because the geese travel by foot with the goslings. Failed breeders have a different molting site outside of Finland in Novaya Zemlya (Piironen et al., 2021), and their molted feathers were not collected in this study. The samples collected in the present study are hereafter referred to as the “Finnish population.” We used mostly a citizen‐science approach—in which the public is involved in scientific research—to collect feather samples. As single feathers cannot be aged reliably, our sample might include adults, juveniles, and goslings with unknown proportions. We also created outgroups for our Finnish population by obtaining samples of: (1) Swedish breeding taiga bean geese (2014; *n* = 7), (2) migrating Russian taiga bean geese hunted from Finland (Honka et al., 2017) or Estonia (2010–2012, 2017; *n* = 7), (3) Norwegian breeding tundra bean geese (2002, 2006; *n* = 7), and (4) Iceland breeding pink‐footed geese (Ruokonen et al., 2005, the Natural History Museum of Reykjavik; *n* = 7) (Figure 3). All feathers were stored in paper envelopes at room temperature prior to DNA extraction. Sampling of feathers was performed in a laboratory in which no PCR products are handled.

DNA from a calamus and from a blood clot (when visible; Horváth et al., 2005) was extracted using QuickExtract DNA Extraction Solution (Lucigen) according to the manufacturer's protocol, except for a 15 min incubation at 65 ⁰C instead of 6 min. Feathers showing very poor preservation and clear environmental exposure (brittle and discolored calamus) were omitted from the DNA extraction. Also, feathers belonging to other bird species based on morphology (e.g., feather shape typical of common crane *Grus grus*) were omitted from DNA extraction. The extracted DNA was stored in −20°C.

### 2.3 Microsatellite genotyping

We genotyped the samples for 28 microsatellite loci (Kleven et al., 2016; Noreikiene et al., 2012) in four multiplex reactions (A–D) (Table S1) using Multiplex PCR kit (Qiagen) in 6 µl reaction volumes according to manufacturer's protocol. The forward primers were fluorescently labeled with 6FAM, NED, PET, or VIC dyes (Table S1). One microliter of template DNA was used in each reaction. The thermal profile consisted of 95°C for 15 min, followed by 40 cycles of 94°C for 30 s, 57°C (panels A and B) or 60°C (panels C and D) for 90 s, and 72°C for 30 s with a final extension of 60°C for 30 min. We performed fragment analysis with an ABI 3730 and scored the alleles using GeneMapper 5 (Applied Biosystems).

We used a stepwise approach to amplify the microsatellite panels. First, we amplified the samples with panel B (five loci) to screen for quality and to reduce the number of duplicate individuals, as follows. We performed individual identification by visual inspection of the genotypes and by using the “Regroup genotypes” option in the program Gimlet v.1.3.3. (Valière, 2002). If one sampling site included several identical genotypes, these were assumed to be replicates of the same individual and all except one was excluded. Next, we amplified panel A (seven loci) from the apparently good‐quality samples selected in the first step. We performed the individual identification similarly as after the panel B, as genotyping errors could have been interpreted as different individuals in the first step and again excluded all but one identical genotype (taking into account the genotyping errors). Panels C and D were amplified with the samples selected in the second step and the individual identification was performed once more in order to test for any remaining errors in the data and again kept only one of the identical genotypes.

Due to the inherent presence of allele dropouts and false alleles in microsatellite data (Taberlet et al., 1999), we regarded multilocus genotypes with up to three mismatches to belong to the same individual. Mismatches in 4–5 loci between multilocus genotypes were carefully checked and if the pattern was consistent with allele dropout due to poor‐quality template DNA, the samples were excluded. Consensus genotypes for each individual were created manually by choosing the majority genotype for each locus or, if only two replicates were genotyped from an individual, the heterozygote, as allele dropouts were more likely than false alleles (see Table S1). In addition, individuals with more than 25% missing data were excluded, resulting with 491 individuals. Three individuals were excluded from the analysis of the taiga bean geese because these samples had an mtDNA sequence of a different subspecies or were possibly still in spring migration (see below). Thus, 488 individuals were included in analyses unless otherwise stated. We calculated the unbiased probability of identity (*P*_ID_) and probability of identity of siblings (*P*_ID SIB_) (Taberlet & Luikart, 1999) from the identified individuals using the Gimlet program.

### 2.4 Molecular sexing

Sexing of the individuals was based on fluorescently labeled (6FAM) forward primer ASW12‐D3 (Guzzetti et al., 2008) and reverse primer HZW278 (Gravley et al., 2017) targeting the HINTZ/W gametologs. We performed the PCR reactions and fragment analyses similarly as for the microsatellites except the annealing temperature was 50°C and the final extension was performed at 72°C. These markers produce a 287 bp (base‐pair) fragment in females and a 297 bp fragment in males (including the primer sequences). The sexing results were verified using seven individuals from which the sex was inspected by cloacal examination during goose ringing. In addition, we used a Chi‐squared test to test if the number of females and males differed from the expectation of equal numbers. Bean geese form stable pair bonds and both parents participate in the rearing of goslings, so an equal number of females and males would be expected.

### 2.5 Microsatellite analyses

The data were divided into four geographical regions (Figure 3) and all analyses were performed on these groups. We used the program Micro‐Checker v. 2.2.3 (van Oosterhout et al., 2004) to assess the accuracy of the microsatellite typing and the program FreeNA (Chapuis & Estoup, 2007) to calculate the null‐allele rate. A custom program (Microsat_errcalc; Honka & Merikanto, 2020) was used to calculate allele dropout (ADO) and false‐allele (FA) rates from different feather samples belonging to the same individual. Number of alleles (*A*), number of private alleles (*PA*), observed heterozygosity (*H*_O_), expected heterozygosity (*H*_E_), and inbreeding coefficient (*F*_IS_) were calculated using the program GenAlEx 6.503 (Peakall & Smouse, 2006, 2012), and allelic richness (*A*_R_) was calculated using the program FSTAT 2.9.4 (Goudet, 1995). Departure from the Hardy–Weinberg equilibrium (HWE) and the degree of linkage disequilibrium (LD) were determined using the program Genepop 4.7.0 (Rousset, 2008) with a sequential Bonferroni correction (Rice, 1989) applied to these tests. We evaluated the power of the microsatellite markers to detect a signal of population differentiation by using the simulation program POWERSIM 4.1 (Ryman & Palm, 2006).

We inferred parentage and sibships using the program Colony 2.0.6.5 (Jones & Wang, 2010) by setting “monogamy” for both females and males (as geese are known to form stable pair bonds) and performed three iterations of the long run with inbreeding model with a full‐likelihood method. All taiga bean goose individuals (*n* = 488) were placed in the candidate offspring category, females (*n* = 237) and geese of unknown sex (*n* = 62) (based on molecular sexing) were placed in the candidate mother category (total *n* = 299), and males (*n* = 189) and geese with unknown sex (*n =* 62) (based on molecular sexing) were placed in the candidate father category (total *n* = 251). We excluded each individual from being the mother or father of itself. We used the calculated ADO and FA rates (Table S1) as the marker error rates. We calculated relatedness (*r*) between goose dyads using the program ML‐relate (Kalinowski et al., 2006) taking account of the null alleles. As it has been shown that sampling of close relatives biases genetic structure analyses, especially in the program Structure (Anderson & Dunham, 2008; Rodríguez‐Ramilo & Wang, 2012), we created a dataset from which the inferred parents, all but one sibling and individuals with *r* > 0.55, were removed resulting in a subset of data with 376 non‐kin individuals. All analyses were performed on this non‐kin dataset except for *F*_ST_ (fixation index), *ɸ*_ST_, and *N*_e_ (effective population size) that were calculated with the full dataset because the precision of these genetic estimates may suffer with the purging of all siblings (Waples & Anderson, 2017). In addition, analyses in the program Structure (Pritchard et al., 2000) were performed for both the non‐kin and the full dataset. Due to complex nature of our data collected by a citizen‐science approach without knowledge of the identity of the samples—thereby potentially including multiple cohorts and a family structure—our data should be considered non‐random and family correlated.

We inferred population structure using the program Structure 2.3.4. (Falush et al., 2003; Hubisz et al., 2009; Pritchard et al., 2000) with the LOCPRIOR option, with individuals within 16 km treated as coming from one location. We used an admixture ancestry model and correlated allele frequencies with a burn‐in of 100,000 and a run length of 1,000,000. Five iterations were performed with the possible number of clusters (*K*) set from 1 to 10. The ad hoc approach of Evanno et al. (2005) was used to infer the most likely number of *K* clusters in the data as implemented in the program Structure Harvester (Earl & vonHoldt, 2012). We used the program Clumpak 1.1. (Kopelman et al., 2015) to visualize the Structure results and to create consensus among the different iterations. In addition, we used Discriminant Analysis of Principal Components (DAPC; Jombart et al., 2010) implemented in the R package (R Core Team, 2018) “adegenet” 2.1.1 (Jombart, 2008; Jombart & Ahmed, 2011) to assess population structure. The lowest number of non‐kin samples was in the Western Finland region (*n* = 50) and, accordingly, we randomly chose an equal number of samples in each geographical region (*n* = 50 in each population, total *n* = 200) as the DAPC could be biased due to unequal samples sizes. Cross‐validation (xval command) was used to evaluate the number of PCs to retain. DAPC was also used to compare the outgroup samples and the Finnish population—by performing DAPC first on the outgroups and then importing the “unknown” Finnish population into this same framework using the “predict” function. Pairwise *F*_ST_‐values were calculated using the ENA correction implemented in the program FreeNA due to the possible presence of null alleles in the data. To test for isolation by distance, we performed a Mantel test implemented in the R package (R core team, 2018) “ade4” 1.7.13 (Bougeard & Dray, 2018; Chessel et al., 2004; Dray & Dufour, 2007; Dray et al., 2007) using Euclidean distances. We also performed a spatial autocorrelation analysis (Smouse & Peakall, 1999) using the program GenAlEx to test for fine‐scale geographic patterns. The significance of the analysis was tested using a heterogeneity test (Smouse et al., 2008). In addition, the autocorrelation analysis was performed on females and males separately to estimate sex biases in relatedness (Banks & Peakall, 2012).

The effective population size (*N*_e_) was estimated with a linkage disequilibrium model (Hill, 1981; Waples, 2006; Waples & Do, 2010), assuming monogamy and using 0.05 as the critical value for allele frequency as implemented in the program NeEstimator v2.1 (Do et al., 2014). The *N*_e_ estimate was compared with the estimate based on sibship assignment (Wang, 2009) by the program Colony. The presence of historical bottlenecks was evaluated with the program Migraine 0.5.4 (Leblois et al., 2014) using a single population with a past population size change (OnePopVarSize) option and Generalized Stepwise Mutation (GSM; Pritchard et al., 1999) model. Migraine was run with 500 points and 2000 runs per point with seven iterations. Ancestral population size (*N*_anc_), current population size (*N*), and time of demographic change in generations (*T*) were resolved from scaled parameters produced by Migraine (*θ*_anc_ = 2*N*_anc_ *µ*, *θ* = 2*Nµ* and *D* = *T*/2*N*) by assuming a microsatellite mutation rate of *µ* = 5 × 10^−4^ per locus per generation (Dib et al., 1996; Ellegren, 2000; Sun et al., 2012) and a generation time of 5–7.5 years (Dillingham, 2010). The demographic change was evaluated using a parameter called *N*_ratio_ (*N*/*N*_anc_), with *N*_ratio_ > 1 indicating a population growth and *N*_ratio_ < 1 indicating a population bottleneck. The statistical significance was evaluated using 95% confidence intervals. If the 95% confidence intervals do not span 1, the results are statistically significant (Leblois et al., 2014). For other tests of possible bottlenecks, see Supplementary text 1.

We studied hybridization between the taiga bean goose and either the pink‐footed goose (taiga bean goose x pink‐footed goose) or the tundra bean goose (taiga bean goose x tundra bean goose) by using a simulation study. First, we simulated 100 pure parental individuals (selected by *q *> 0.99 by a preliminary NewHybrids run) and 100 individuals in different hybrid classes (F1, F2, and backcrosses) using the program HybridLab1.0 (Nielsen et al., 2006). We used these simulated individuals as an input for the program NewHybrids 1.1 (Anderson & Thompson, 2002) with Jeffreys‐like priors for both mixing proportions and allele frequencies, setting burn‐in to 20,000 sweeps and the chain length to 100,000 MCMC sweeps with the z option. We compared these results to the Structure program run with the admixture ancestry model and correlated allele frequencies with burn‐in set to 10,000 and run length to 100,000. Five iterations were performed with the number of *K* set to 2. As the Structure program performed better than the NewHybrids program (Figures S1–S4), we secondly run Structure analysis with the simulated parentals and the Finnish population including also the two tundra bean geese and the plausible pink‐footed goose individual (*n* = 491). In this second analysis, the aim was to search for admixture with other subspecies or species in the Finnish population.

### 2.6 Mitochondrial DNA analyses

The hypervariable portion of the mitochondrial control region domain I (210 bp) was amplified using primers AdCR1F and AdCR2R (Honka et al., 2018) from the individuals identified with microsatellite genotyping (*n =* 491). The primers were designed to contain mismatches to Numts (nuclear sequences of mitochondrial origin; Lopez et al., 1994), which are problematic in genetic studies if not accounted for (Bensasson et al., 2001; Sorenson & Quinn, 1998). The PCR reactions were performed in 10 µl volumes using 1 x Phusion HF buffer (Thermo Fisher Scientific), 0.2 mM of each dNTPs, 0.5 µM of forward and reverse primers, 0.02 U/µl of Phusion DNA Polymerase (Thermo Fisher Scientific), and 1 µl of template DNA. The thermocycling conditions were 98°C for 4 min, followed by 40 cycles of 98°C for 30 s, 57°C for 30 s, and 72°C 40 s with a final extension of 72°C for 7 min. Double‐stranded sequencing with the PCR primers was performed using BigDye Terminator v.3.1 (Applied Biosystems) and the reactions were run on an ABI3730.

The sequences were manually edited using the program CodonCode Aligner v.4.0.4. (CodonCode Corporation) and aligned with GenBank sequences of bean geese (accession numbers: EU186807–EU186812, EU186827, AF159951, and MH491806–MH491819; Ruokonen et al., 2000; Ruokonen et al., 2008; Honka et al., 2017), pink‐footed geese (AF159952–AF159953; Ruokonen et al., 2000), and greylag geese (*Anser anser*) as an outgroup (AF159961; Ruokonen et al., 2000) using the program BioEdit 7.2.5 (Hall, 1999). A median joining network (Bandelt et al., 1999) was constructed using the program PopART (Leigh & Bryant, 2015), and number of haplotypes (*H*), haplotype (*h*) and nucleotide (*π*) diversities, Tajima's *D* (*D*), and Fu's Fs (*Fs*) were calculated using program DnaSP v. 6.12 (Rozas et al., 2017). The presence of genetic structure on individual and region levels was tested by an analysis of variance framework using analysis of molecular variance (AMOVA), which is based on hierarchical variance of gene frequencies. We calculated pairwise *ɸ*_ST_‐values between regions and performed an AMOVA analysis using the program Arlequin 3.5.2.2. (Excoffier & Lischer, 2010) with the substitution model of Jukes–Cantor (Jukes & Cantor, 1969). This was the best substitution model based on Akaike information criteria (AIC) and Bayesian information criterion (BIC) values in the program MEGA X (Kumar et al., 2018) and is supported in the Arlequin program. A sequential Bonferroni correction (Rice, 1989) was applied to the *ɸ*_ST_ statistics.

## 3 RESULTS

### 3.1 Microsatellite genotyping success

Microsatellite genotyping was successful for 1805 feather samples (at least one multiplex panel successful) with a success rate of 84%. After omitting possible redundancy, this amounted to a total of 491 individuals in our dataset. One individual that may have been on spring migration and had pink‐footed goose mtDNA (individual found dead, see Figure 3) was omitted from the Finnish breeding population analyses but included in the mtDNA haplotype network, maps, and hybridization analyses. Similarly, we also omitted two tundra bean goose individuals, leaving us with a final total of 488 taiga bean goose individuals. The error rate in the whole dataset (28 loci) was 0.019 per allele over all loci and 0.039 per locus over all loci. The mean ADO rate was 0.041, the mean FA rate was 0.009, and the mean null allele rate was 0.034 (Table S1). The cumulative unbiased probability of identity and the cumulative probability of identity of siblings were low (*P*_ID_ = 5e−25, *P*_ID SIB_ = 4e−10; Table S1), indicating that our markers can separate different individuals with high confidence.

No evidence of large allele dropout was present in the loci, but the program MicroChecker suggested that some loci show stuttering. However, the stuttering was not consistent over the geographical regions (see Figure 3 for the regions). We removed the loci Abra14 and Abra29 due to low polymorphism, Abra9 due to a high rate of missing data (43%) and Abra15 and Abra43 due to a high frequency of null alleles (>10%) (Table S1). Locus Afa18 was not in Hardy–Weinberg equilibrium in any of the four geographical regions and was removed. Therefore, a final total of 22 loci were kept for further analyses. Several other loci showed deviations from Hardy–Weinberg equilibrium and some were in linkage disequilibrium after Bonferroni correction, but these were not consistent between regions, so these loci were kept in the analyses. Using these 22 loci, their frequency distributions, and the numbers sampled in each region, the simulation program POWSIM indicated that the power of the markers is fairly high. For example, a true *F*_ST_ of 0.001 would be detected with a probability of 100%, indicating that these loci are suitable for detecting significant divergence, had it occurred.

### 3.2 Molecular sexing and relatedness

Molecular sexing using the HINTZ/W gametologs showed congruent results with the cloacal examination except for one goose out of seven. Based on the cloaca, this goose was assigned as a female while based on molecular sexing it was a male. This sample was replicated and the same result was obtained by molecular sexing. We identified 237 females and 189 males in the Finnish population which was significantly different from equality (*χ* ^2^ = 5.40, *P* = 0.02). The sex could not be determined for 62 individuals due to lack of amplification of this marker.

Most of the geese dyads studied were unrelated (80,576; relatedness value 0.00–0.04), 38,049 dyads had relatedness values between 0.05 and 0.39 and 131 geese dyads had relatedness values between 0.40 and 0.59 indicating potentially parent–offspring or sibling relationships according to the ML‐relate program. Relatedness values of approximately 0.50 were found for all known parent–offspring and sibling dyads of ringed geese (two sibling dyads and one parent offspring dyad), except for one gosling that could not have been offspring of the parents it was captured with (unrelated to the mother, *r =* 0.25, between the father and the gosling). We identified 23 candidate fathers and 28 candidate mothers with probabilities over 0.90.

We identified two male geese harboring mtDNA of the pink‐footed goose to have sired offspring with females having taiga bean goose mtDNA. Fifty‐two pairs of full siblings were identified with probabilities of 1.00, these also shared mtDNA haplotypes. Seventy‐two geese dyads showed relatedness values between 0.60 and 0.86, suggesting potential inbreeding. Based on these results, we created a subset of the data that did not contain parent–offspring or sibling relationships or highly related individuals (*r* > 0.55), producing a final dataset of 376 non‐kin individuals.

### 3.3 Genetic diversity

The number of alleles varied between 171 and 194 with a mean of 184 over the geographical regions (Table 1). Allelic richness, which takes into account the sample size, was almost the same in all regions (*A*_R_ = 7.4–7.6; Table 1). The Eastern Finland/Kainuu region had the largest number of private alleles (*PA* = 9), followed by Lapland (*PA* = 7; Table 1). Observed and expected heterozygosities were similar over the regions (mean *H*_O_ = 0.64 and mean *H*_E_ = 0.69) with all regions showing higher expected than observed heterozygosities (Table 1). Inbreeding coefficients (*F*_IS_) ranged between 0.05 and 0.10 (Table 1). All regions showed deviation from the Hardy–Weinberg equilibrium when summed over loci (Fisher's exact test; Table 1).

**TABLE 1:** Summary statistics for the 376 non‐kin taiga bean geese (*Anser fabalis fabalis*) genotyped by 22 microsatellite loci and grouped by four geographical regions (see Figure 3)

Region	*n*	*A*	*A*_R_	*PA*	*H*_O_	*H*_E_	*F*_IS_	*P*_HWE_
Western Finland	50	171	7.5	3	0.64 (0.03)	0.69 (0.03)	0.07 (0.03)	**0.00**
Eastern Finland/Kainuu	140	194	7.6	9	0.64 (0.03)	0.70 (0.03)	0.07 (0.02)	**0.00**
Northern Ostrobothnia/Southern Lapland	87	183	7.6	4	0.66 (0.04)	0.69 (0.03)	0.05 (0.02)	**0.00**
Lapland	99	186	7.4	7	0.62 (0.04)	0.69 (0.03)	0.10 (0.02)	**0.00**
Mean over regions		184	7.5	6	0.64 (0.02)	0.69 (0.02)	0.07 (0.01)	**0.00**

Note

Sample size (*n*), total number of alleles (*A*), allelic richness (*A*_R_), number of private alleles (*PA*), observed heterozygosity (*H*_O_), expected heterozygosity (*H*_E_), inbreeding coefficient (*F*_IS_), and *P*‐value from test for deviation from the Hardy–Weinberg equilibrium (*P*_HWE_). Statistically significant *P*‐values after Bonferroni correction shown in bold. Standard errors are in parentheses.

### 3.4 Population structure

The Structure analyses indicated little difference between the regions (mapped in Figure 3) or indeed between any individuals within the Finnish breeding taiga bean goose population (Figure S5a), with some structure introduced when using the full dataset including kin (Figure S5b). The optimal *K* was 4 based on the ad hoc statistics Δ*K*, but the log likelihood values did not reach a clear plateau (Figure S6), instead all samples were assigned to all clusters with high admixture proportions indicating that the *K* would actually be 1.

**FIGURE 4:**
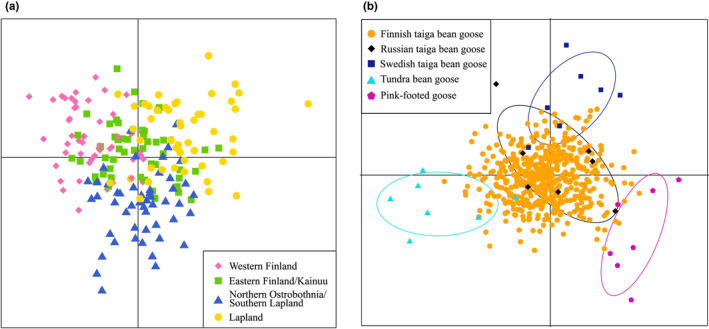
Discriminant analysis of principal components (DAPC) for Finnish breeding taiga bean geese (*Anser fabalis fabalis*) and outgroups (Russian and Swedish taiga bean goose, Norwegian tundra bean goose [*Anser fabalis rossicus*], and Icelandic pink‐footed goose [*Anser brachyrhynchus*]). (a) DAPC for Finnish breeding taiga bean geese with an equalized number of samples per geographic region (*n* = 50 per region, chosen randomly except for the Western Finland region which contained 50 non‐kin samples; as mapped in Figure 3). Number of PCs = 80. (b) DAPC for outgroups: Russian and Swedish taiga bean goose, Norwegian tundra bean goose and Icelandic pink‐footed goose. The Finnish breeding taiga bean goose samples (Finland) were fitted to this DAPC analysis as “unknown” samples in order to identify the clustering of the Finnish samples without assuming any prior genetic group. Number of PCs = 12. Inertia ellipses represent graphical summaries of a cloud of points

The DAPC analyses showed that slight genetic structuring might be present between Western Finland and Lapland regions, as the samples mostly did not overlap (Figure 4a). Samples from Northern Ostrobothnia/Southern Lapland and Eastern Finland/Kainuu region, on the other hand, overlapped with the other two regions, although several samples from Northern Ostrobothnia/Southern Lapland were distinct from the other populations (Figure 4a). The DAPC analysis performed to the outgroups showed that all outgroups (Swedish taiga bean geese, Russian taiga bean geese, tundra bean geese, and pink‐footed geese) clustered to their own groups except for minor overlap between Swedish and Russian taiga bean geese (Figure 4b).

When the Finnish samples were fitted to this framework, most of the samples clustered with the Russian taiga bean geese. However, some samples showed genetic affinity to Swedish taiga bean geese, tundra bean geese, and to a smaller extent, to the pink‐footed geese (Figure 4b).

Pairwise *F*_ST_‐values were always very low, with mean values between regions of less than 0.005 (Table 2). The Western Finland region was the most differentiated from all of the other regions, although none of the values were statistically significant.

**TABLE 2:** Pairwise *F*_ST_‐ and *ɸ*_ST_‐values among Finnish taiga bean goose (*Anser fabalis fabalis*)

	Western Finland	Eastern Finland/Kainuu	Northern Ostrobothnia/Southern Lapland	Lapland
Western Finland	—	0.0228 (0.0004)	0.1977* (0.1481*)	0.0045 (−0.0047)
Eastern Finland/Kainuu	0.0032 (0.0024)	—	0.0779* (0.0855*)	0.0000 (−0.0081)
Northern Ostrobothnia/Southern Lapland	0.0046 (0.0028)	0.0016 (0.0004)	—	0.1107* (0.0951*)
Lapland	0.0042 (0.0022)	0.0026 (0.0008)	0.0025 (0.0007)	—

Note

Pairwise *F*_ST_‐values for microsatellite data below the diagonal among 488 Finnish taiga bean goose grouped by geographical regions (see Figure 3) with non‐kin individuals in parentheses (*n* = 375). Pairwise *ɸ*_ST_‐values for mitochondrial data (*n* = 447) above diagonal with non‐kin individuals in parentheses (*n* = 343). Statistically significant values after Bonferroni correction indicated with an asterisk.

**FIGURE 5:**
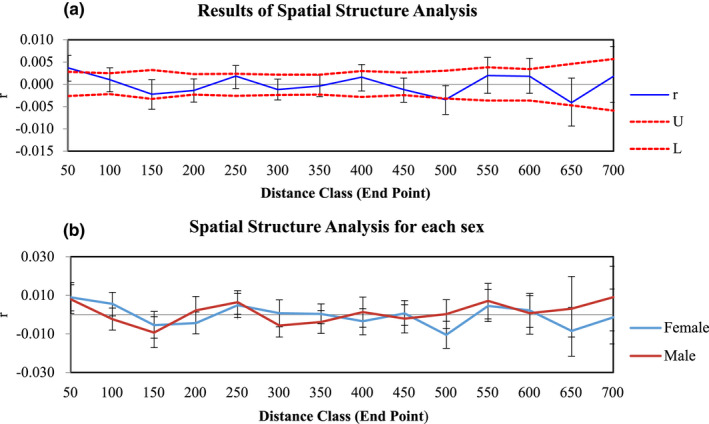
Correlogram from spatial autocorrelation analysis for (a) non‐related taiga bean geese (*Anser fabalis fabalis*; *n* = 376) and (b) females and males separately (*n =* 328). The autocorrelation coefficient (*r*) was plotted against the function of distance class (50 km). The 95% confidence interval (dashed lines, U = upper limit, L = lower limit) was determined by 999 permutations with the null hypothesis of no population structure, and 95% error bars were determined by bootstrap resampling of 1000 replicates

We found no isolation‐by‐distance pattern using the Mantel test (*r =* 0.019, *P* = 0.18; Figure S7) or the spatial autocorrelation analysis (Figure 5a). No sex‐specific differences were evident in the spatial autocorrelation analysis either (Figure 5b).

### 3.5 Effective population size and bottlenecks

The effective population size (*N*_e_) of the full dataset including kin was estimated to be 1127.6 individuals (95% confidence intervals CI: 937.2–1392.9) using the linkage disequilibrium method. The *N*_e_ estimate based on sibship using the full likelihood method was 1134 individuals (95% CI: 927–1327). No signs of recent population bottlenecks were detected, instead, the Migraine analysis indicated population growth as the *N*_ratio_ was 7.8 (CI: 1.746–21.24) and the 95% confidence did not overlap with 1. The Migraine analysis indicated a population growth starting from 21,766 to 32,648 years ago (assuming generation time 5–7.5 years; *T* = 4353 generations, 95% CI: 1138–13,511) from 601 individuals (*N*_anc_, 95% CI: 255–2490) to the current census population size of 4701 individuals (*N*, 95% CI: 3790–5876) (Figure S8).

### 3.6 Hybridization

**FIGURE 6:**
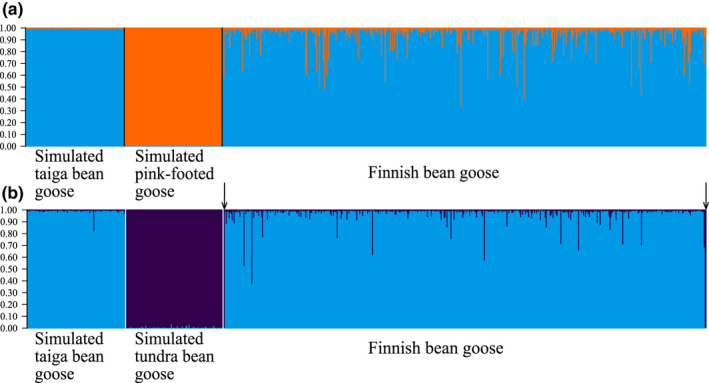
Structure plot with *K* = 2 for: (a) simulated taiga bean goose (*Anser fabalis fabalis*), simulated pink‐footed goose (*Anser brachyrhynchus*), and bean goose breeding in Finland; (b) simulated taiga bean goose, simulated tundra bean goose (*Anser fabalis rossicus*), and bean goose breeding in Finland. Two tundra bean goose individuals identified using mitochondrial DNA indicated in the Finnish dataset with an arrow. These appear as tundra bean geese based on their microsatellites as well

The NewHybrids and the Structure analysis based on simulated data showed that identification of different hybrid categories (F1, F2, and backcrosses) between the taiga bean and the pink‐footed goose and between the taiga bean and the tundra bean goose is difficult using microsatellite markers due to the extent of variation within each hybrid category (Figures S1–S4). However, the pure simulated parentals were readily identifiable. We also run a Structure analysis using the simulated parental populations and the full dataset of the Finnish population including the three individuals omitted from other analyses (two Finnish tundra bean geese and the goose found dead (see Figure 3); *n =* 491). This analysis showed that the simulated pure taiga bean geese and pink‐footed geese could be separated and that some Finnish taiga bean geese showed admixture with pink‐footed geese (Figure 6a). No pure pink‐footed geese were present in our dataset according to this analysis. The analysis for the simulated taiga and tundra bean geese showed similar results. Some of the Finnish taiga bean geese showed admixture with tundra bean geese (Figure 6b). The two tundra bean geese sampled from Finland in this study were identified as pure tundra bean geese with >0.99 probability (Figure 6b, arrows).

### 3.7 Mitochondrial DNA

**FIGURE 7:**
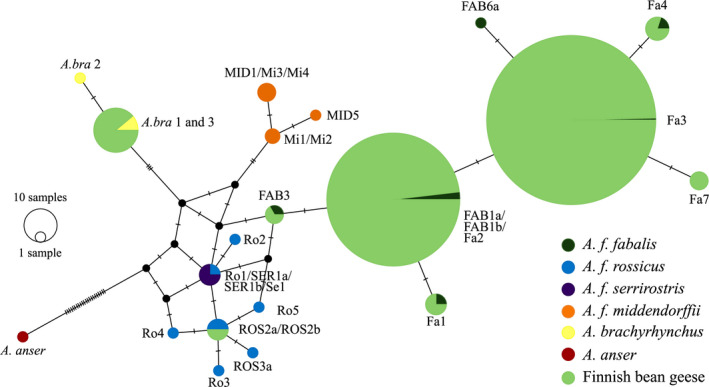
Median joining haplotype network for the Finnish breeding bean geese, different bean goose subspecies (*Anser fabalis fabalis*, *Anser fabalis rossicus*, *Anser fabalis serrirostris*, and *Anser fabalis middendorffii*), and pink‐footed goose (*Anser brachyrhynchus*). An mtDNA sequence of greylag goose (*Anser anser*) was used to root the haplotype network. The sizes of the circles are proportional to the frequency of each haplotype and tick marks across branches indicate the number of mutational differences. Forward slashes between haplotype names denote identical haplotypes based on the sequenced fragment but differing based on the whole control region sequence

**FIGURE 8:**
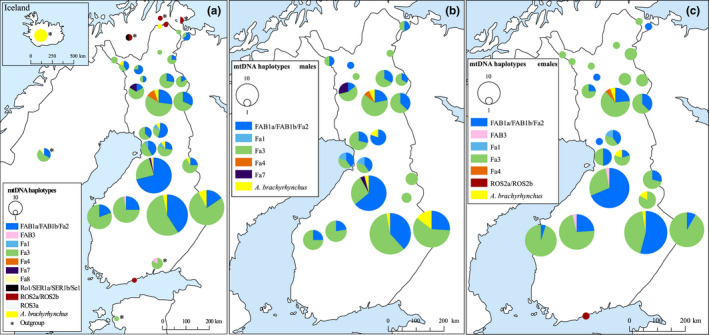
Mitochondrial haplotypes of all the Finnish bean geese (*Anser fabalis*), only males or females, and outgroup samples (indicated with an asterisk) showed on a map as pie charts. (a) All Finnish taiga bean geese and outgroups. Figure insert shows Iceland. The outgroup in southeastern Finland and Estonia consists of Russian taiga bean geese (*Anser fabalis fabalis*), hunted along their migration route. The outgroup in Sweden consists of breeding or molting taiga bean geese, the outgroup in Norway consists of breeding or molting tundra bean geese (*Anser fabalis rossicus*), and the outgroup in Iceland consists of museum feathers of pink‐footed goose (*Anser brachyrhynchus*). (b) Male taiga bean geese. (c) Female taiga bean geese. The size of the circles corresponds to the frequency of the haplotype. Close sample sites were merged for better visualization of the data

We successfully sequenced a 210‐bp‐long fragment of the mtDNA control region from 446 samples. Thirteen samples were identified as Numts and excluded. The vast majority of the studied individuals possessed *A. f. fabalis* mtDNA haplotypes (*n* = 432; Figure 7). The most common haplotype among the Finnish bean geese was Fa3 (*n =* 261), followed by the haplotype FAB1a/FAB1b/Fa2 (*n* = 159). The slashes between haplotype names denote identical haplotypes based on the sequenced region. These haplotypes, however, differ based on the whole control region (Honka et al., 2017; Ruokonen et al., 2008). The two most common haplotypes were distributed throughout Finland (Figure 8a,b,c). Four rarer haplotypes were also found: FAB3 (*n =* 2), Fa1 (*n =* 3), Fa4 (*n =* 4), and Fa7 (*n =* 3; GenBank accession number: MT023340). The rarer haplotypes were more localized with haplotype Fa1 only found in Northern Ostrobothnia, Fa4 only in Southern Lapland, Fa7 in only males in two areas in Northern Ostrobothnia and Lapland, and FAB3 only in females from Ostrobothnia (Figure 8a–c). Haplotype FAB3 was also common among the Russian geese, which were sampled along their migration route from southeastern Finland (Figure 8a). Haplotypes Fa3, FAB1a/FAB1b/Fa2, and Fa8 (GenBank accession number: MT023341) were found among the Swedish taiga bean geese (Figure 8a).

Surprisingly, 16 individuals possessed an mtDNA haplotype typical of the pink‐footed goose (Figure 7) and these individuals were distributed throughout Finland (Figure 8a–c). Two individuals had an mtDNA belonging to the tundra bean goose *A. f. rossicus* (Figure 7). One of these was found from the Helsinki metropolitan area, which is far outside the natural breeding range of either subspecies (Figure 8a,c). The other was found in northernmost Lapland (Figure 8a). The Norwegian outgroup consisted solely of the tundra bean geese (Figure 8a).

The number of haplotypes was highest in the Northern Ostrobothnia/Southern Lapland region and lowest in the Western and the Eastern Finland/Kainuu regions (Table 3). Haplotype and nucleotide diversities were lowest in the Western Finland region and highest in the Northern Ostrobothnia/Southern Lapland region (Table 3). Tajima's *D*‐value was negative in all of the regions while Fu's Fs was negative only in the Northern Ostrobothnia/Southern Lapland and the Lapland regions (Table 3). Pairwise *ɸ*_ST_‐values were either zero or very close to zero in all comparisons except between the Northern Ostrobothnia/Southern Lapland region and all the other regions (Table 2). The pairwise *ɸ*_ST_‐values were even lower if the non‐kin dataset was used, with some values even below 0 (Table 2). Thus, the Northern Ostrobothnia/Southern Lapland region was moderately genetically differentiated (*ɸ*_ST_ = 0.11–0.20). The pairwise *ɸ*_ST_‐values were higher for females (*ɸ*_ST_‐values between 0.00 and 0.27) than in males (*ɸ*_ST_‐values between 0.00 and 0.13) (Table 4). In addition, 93.8% of the total variation was within region variation and 6.2% was among region variation (*ɸ*_ST_ = 0.062; *P* < .001) according to AMOVA.

**TABLE 3:** Genetic variability in the hypervariable portion of the mitochondrial control region domain I (210 base‐pairs) among non‐kin Finnish taiga bean geese (*Anser fabalis fabalis*, *n* = 342)

Region	*n*	*H*	*h* (SD)	*π* (SD)	*D*	*Fs*
Western Finland	47	3	0.399 (0.069)	0.0021 (0.0004)	−0.087 (NS)	0.038 (NS)
Eastern Finland/Kainuu	122	3	0.464 (0.036)	0.0039 (0.0009)	−0.828 (NS)	2.243 (NS)
Northern Ostrobothnia/Southern Lapland	84	6	0.577 (0.035)	0.0048 (0.0011)	−0.934 (NS)	−0.527 (NS)
Lapland	90	5	0.499 (0.042)	0.0037 (0.0009)	−1.433 (NS)	−0.342 (NS)
Finland	343	7	0.519 (0.018)	0.0040 (0.0005)	−0.864 (NS)	−0.789 (NS)

Note

Number of samples (*n*), number of haplotypes (*H*), haplotype diversity (*h*), nucleotide diversity (*π*), Tajima's *D* (*D*), and Fu's Fs (*Fs*). NS: non‐significant values. The different geographical regions are mapped in Figure 3.

**TABLE 4:** Pairwise *ɸ*_ST_‐values among female and male Finnish taiga bean goose (*Anser fabalis fabalis*) for mitochondrial data

	Western Finland	Eastern Finland/Kainuu	Northern Ostrobothnia/Southern Lapland	Lapland
Western Finland	—	−0.0005	0.1267*	−0.0241
Eastern Finland/Kainuu	0.0293	—	0.0462*	−0.0065
Northern Ostrobothnia/Southern Lapland	0.2689*	0.08768*	—	0.0753*
Lapland	−0.0079	−0.0048	0.1664*	—

Note

Pairwise *ɸ*_ST_‐values for females below diagonal (*n* = 218) and for males above diagonal (*n =* 174). Statistically significant values after Bonferroni correction are indicated with an asterisk.

## 4 DISCUSSION

We did not detect clear population structure within the Finnish breeding taiga bean geese using microsatellite markers. All analyses suggested close to a panmictic population, except the DAPC that indicated slight structuring among Western Finland, Northern Ostrobothnia/Southern Lapland, and Lapland (see Figure 3). Presence of geographically localized mtDNA haplotypes and higher *ɸ*_ST_‐values for females than in males, however, suggested at least some maternal genetic structure. It was unforeseen to find such little genetic structure within such a large geographic area, but the pairing system of geese, in which pair formation occurs already in common wintering or spring staging areas, can explain these results. We found moderate genetic diversity and signs of inbreeding within the Finnish taiga goose population.

Surprisingly, we also found that a pink‐footed goose mtDNA haplotype is widespread (although at low frequency) in the taiga bean goose. This could indicate hybridization between the taiga bean goose and the pink‐footed goose and admixture was also evident in the microsatellite data. In addition, we confirmed breeding of tundra bean geese in the northernmost Lapland and the presence of a vagrant tundra bean goose in Southern Finland. The microsatellite data suggested introgression between the taiga and the tundra bean goose as well.

### 4.1 Citizen science

This study proved that a citizen‐science approach to feather collection was highly efficient for the elusive bean goose. About 89% of the samples were collected by a citizen‐science approach, while the rest were collected by persons with an affiliation to a research institute. Even though bean goose feathers, especially the down feathers, can be mixed with other large birds in similar habitats, such as the common crane (*Grus grus*) and the whooper swan (*Cygnus cygnus*), only a few feathers had to be excluded as belonging to other species. This was determined either based on feather morphology or because no PCR product amplified from pristine feathers not identifiable based on morphology. Benefits of citizen‐science approach for this study included the large number of samples and the broad geographic range of sampling.

### 4.2 Relatedness, inbreeding, and genetic diversity

Interestingly, we identified one family in which one of the goslings was not the offspring of the social parents, thus representing a possible case of gosling adoption or intraspecific nest parasitism, behaviors which have been observed among other goose species (Zicus, 1981; Weigmann & Lamprecht, 1991; Choudhury et al., 1993; Larsson et al., 1995; Nilsson & Kampe‐Persson, 2003; Anderholm et al., 2009; for review, see Kalmbach, 2006).

Overall, a large majority of the individual goose dyads were unrelated or related to a very low degree. However, positive inbreeding coefficients (*F*_IS_) were detected and a few of the goose dyads showed high relatedness values (*r* = 0.60–0.86), indicating potential inbreeding. This was surprising as the detrimental effects of inbreeding to individual fitness, known as the inbreeding depression, are widely documented (Keller & Waller, 2002), and usually animals avoid inbreeding through several mechanisms (Pusey & Wolf, 1996). However, a single case of sibling pairing has been observed in the Canada goose (*Branta canadensis*; Lebeuf & Giroux, 2013). Heterozygote deficiency, that is, positive *F*_IS_ could be due to Wahlund effect, which is caused by the merging of populations with different allele frequencies (Wahlund, 1928). Even though we detected only a very low level of population structure, it is possible that the population structure is very fine scaled or there is population structure in the wintering sites instead of breeding sites. We were unable to discern exact family relationships beyond parent–offspring and siblings, which might affect the Hardy–Weinberg equilibrium and cause positive *F*_IS_‐values.

Nuclear genetic diversity appeared not to be reduced within the Finnish taiga bean goose population despite the recent decline in population numbers, as the Scandinavian taiga bean goose and other goose species show similar heterozygosity values (de Jong et al., 2019; Ely et al., 2017). However, mtDNA diversity was reduced (*h* = 0.40–0.58) compared to other bean goose subspecies (*h* = 0.68–0.86; Honka et al., 2017) and numerically abundant greater white‐fronted goose (*h* = 0.68–0.96; Ely et al., 2017), but similar in range to the endangered population of lesser white‐fronted goose (*h* = 0.37–0.53; *Anser erythropus*; Ruokonen et al., 2004).

### 4.3 Population structure and demography

We did not observe any population structure when using microsatellite markers with the Structure program as all individuals showed admixture and no genetic clustering was evident for any value of *K*. We found genetic separation among Western Finland, Lapland, and partly in Northern Ostrobothnia/Southern Lapland (see Figure 3) in the DAPC analysis; however, this genetic separation was not strong (Figure 4a). All pairwise *F_ST_*‐values were low (<0.005), showing no clear nuclear differentiation between the geographic regions (Table 2). We did not observe isolation by distance either, further indicating lack of spatial genetic structuring (Figure 5a). These findings indicate that the taiga bean goose population is close to panmictic.

On the other hand, some of the rarer mtDNA haplotypes were localized to certain areas (Figure 8a–c) and the pairwise *ɸ*_ST_‐values showed genetic differentiation (0.11–0.20) between the Northern Ostrobothnia/Southern Lapland region and all other regions (Table 2), implying at least some level of female philopatry. In addition, the pairwise *ɸ*_ST_‐values were higher for females than in males (Table 4), indicating stronger genetic structuring in females than in males. Interestingly, statistically significant *ɸ*_ST_‐values were observed also only in males between the Northern Ostrobothnia/Southern Lapland region and all other regions (Table 4), even though males do not pass on their mitochondria to offspring. This result could imply at least some level of philopatry in males in this region. The reason for this is unknown as Northern Ostrobothnia/Southern Lapland is a central region and thus there should not be restrictions for movement as might be the case for peripheral regions. This region is a core breeding area for the taiga bean goose due to a large number of aapa mires, so potentially this region is a more favorable breeding habitat and thus represents a source population. In addition, an unknown portion of the data could be from juvenile individuals, which might affect the data in this region. Also, we found a difference in haplotype composition between females and males as the FAB3 haplotype was only found in females and haplotype Fa7 only in males. An AMOVA analysis indicated that only about 6.2% of the total variation was between regions, thus mtDNA genetic structuring is still rather limited.

However, nuclear spatial genetic patterns did not indicate sex‐specific differences (Figure 5b). As opposed to male philopatry observed in most other birds, geese show female philopatry to natal areas (Greenwood, 1980; van der Jeugd et al., 2002). Therefore, genetic structure may be promoted especially in the maternally inherited mitochondrial DNA or the female‐specific W chromosome, as was seen here as higher *ɸ*_ST_‐values in females than in males. It seems, however, that the dispersal of male bean geese is so high that it completely homogenizes the nuclear genome. Geese pair in their wintering grounds or in the spring staging areas and males follow females to the female's natal area (Rohwer & Anderson, 1988), which allows geese from even distant breeding areas to pair and thus mediate gene flow in the biparentally inherited nuclear DNA. Long‐distance dispersal of a few males could lead to panmixia as only one migrant per generation in an ideal population is enough to prevent population differentiation due to drift (Mills & Allendorf, 1996; Wang, 2004, but see Vucetich & Waite, 2000). For example, in greylag and brent geese (*Branta bernicla hrota*), most males breed close to their natal site, but a minority of the geese undergo long‐distance dispersal (Harrison et al., 2010; Nilsson & Persson, 2001). On the other hand, a lack of sex‐specific dispersal differences has been observed in Asian breeding swan geese (*Anser cygnoid*; Zhu et al., 2020). The taiga bean geese winter in gregarious flocks mainly in Southern Sweden and Denmark, and to a lesser extent in the Netherlands, western Germany, Poland, and Britain (Nilsson et al., 1999; Figure 2), providing ample opportunities for geese breeding in different areas to mix. Only the taiga bean geese belonging to the Eastern 2 population overwinter in a separate area in Central Asia (Heinicke, 2009; Figure 2), therefore this population could show genetic differentiation.

Studies in other goose species have shown varying levels of genetic structure ranging from a lack of genetic structure (Avise et al., 1992; Harrison et al., 2010; Pellegrino et al., 2015) to phylogeographic clustering (Pujolar et al., 2017; Ruokonen et al., 2004) and strong genetic structuring in brood‐rearing sites (Lecomte et al., 2009). For example, microsatellite studies in the greater white‐fronted goose have discovered a panmictic population with the exception of the Greenland white‐fronted goose (*Anser* *albifrons* *flavirostris*) and the Tule goose (*Anser* *albifrons* *elgasi*) (Ely et al., 2017; Wilson et al., 2018). Also, the different flyway populations of the barnacle goose (*Branta leucopsis*) studied with single nucleotide polymorphism (SNP) markers showed not only genetic structuring but also genetic exchange between all flyways (Jonker et al., 2013). Contrary to our findings in the Finnish breeding bean geese, genetic structuring was observed with microsatellite markers within the taiga bean geese breeding in Central Scandinavia (belonging to the Western flyway management unit, see Figure 2; de Jong et al., 2019). However, de Jong et al. (2019) studied fine‐scale genetic patterns in a geographically restricted area, thus the different scale (family‐level structure in de Jong et al., 2019) of our study could explain the contrasting results.

Both the linkage disequilibrium and the sibship method produced similar estimates of the effective population size with 1128 and 1134 individuals, respectively. The Finnish population size is estimated to be 1700–2500 breeding pairs (i.e., 3400–5000 individuals) based on survey data (Valkama et al., 2011), thus excluding non‐breeders and juveniles. The ratio between the *N*_e_ estimated based on microsatellite markers (this study) and the estimate of the number of breeding individuals is 0.23–0.33. Accordingly, *N*_e_ is just about a quarter or a third of the estimated breeding population. Low *N*_e_/*N* ratios (0.11–0.14) are commonly reported among animals (Frankham, 1995; Palstra & Ruzzante, 2008) and based on *N*_e_ = 1128, the total population size (*N*) would be thus 8057–10,255 individuals, a surprisingly large estimate. However, our estimate of *N*_e_ from only Finnish samples is perhaps somewhat misleading due to a continuous population unrestricted by national borders.

We found no indication of past population bottlenecks, instead, we inferred population expansion starting around 22,000–32,000 years ago. This was unexpected since the taiga bean goose population is in decline (Fox et al., 2010; Fox & Leafloor, 2018). Probably the current population decline is too recent or not severe enough to be detected in genetic bottleneck test. The population expansion starting around 27,000 years ago coincidences with or precedes the Last Glacial Maximum (around 26,500–20,000 years ago; Clark et al., 2009).

### 4.4 Hybridization

We also compared the Finnish population with other taiga bean geese (Russian and Swedish), the tundra bean goose and the pink‐footed goose, using DAPC. As expected, the Finnish geese mostly grouped with the Russian geese (Figure 4b), which should belong to the same flyway management unit (Central; see Figure 2). However, some Swedish geese (Western unit) showed genetic affinity to the Russian and Finnish geese and thus gene flow between the different flyways could be present. Some of the Finnish bean geese clustered closely with either the tundra bean goose or the pink‐footed goose (Figure 6). This could be due to a lack of resolution in our microsatellite markers to discriminate between different populations or an indication of possible hybridization between these populations. Whole‐genome resequencing of the taiga and the tundra bean geese has shown that the genomes of these subspecies are homogenous expect for a few “islands of differentiation” due to extensive gene flow 60,000 years ago (Ottenburghs et al., 2020). Thus, the close affinity of the subspecies is probably due to the past hybridization. Two individuals were shown to be tundra bean geese based on tundra bean goose mtDNA haplotypes (Figure 7) and by microsatellite markers (Figure 6). One of these individuals was sampled from northernmost Finland, confirming that this subspecies breeds in Finland (Figure 8a). The other tundra bean goose was sampled from a metropolitan area in Helsinki (Figure 8a,c), outside of the breeding range of either subspecies and was thus a vagrant bird.

In this study, we showed that the pink‐footed goose mtDNA is widespread (although at low frequency, 4% of the studied population) in the Finnish taiga bean goose population (Figure 8a–c). This was unexpected because the pink‐footed goose breeds in Greenland, Iceland, and Svalbard and no breeding attempts have been recorded in Finland. Although the pink‐footed goose can be found as a vagrant bird in Finland, and has started to regularly migrate through the Western Finland in recent decades (Heldbjerg et al., 2019), we do not believe that we have found 16 pink‐footed geese, but instead admixed individuals. Even though the species do not share breeding grounds, they share common wintering areas in the United Kingdom, Denmark, and the Netherlands, which allow the species to come to contact. The microsatellite data supported admixture (Figure 6), indicating possible hybridization and introgression between the taiga bean goose and the pink‐footed goose. The amount of nuclear admixture seems to be rather high and even more than the 4% suggested by mtDNA. Geese show a high propensity for hybridization (for review, see Ottenburghs et al., 2016), and in the genomics era, increasing number of studies have identified that ancient hybridization, adaptive introgression, and hybrid speciation are much more common than previously thought (Ottenburghs et al., 2017; Taylor & Larson, 2019). Interestingly, the mtDNA of the tundra bean goose had not introgressed into the taiga bean goose according to our results. The mtDNA of the pink‐footed goose may convey adaptive benefits as hybridization often leads to the introgression of adaptive genetic variation (Arnold & Kunte, 2017), but also incomplete lineage sorting can explain the presence of the pink‐footed goose mtDNA (Degnan & Rosenberg, 2009). The pink‐footed goose has been treated as a separate species as it has formed a monophyletic group based on mitochondrial DNA (Ruokonen et al., 2008), although more recently a sister species relationship was suggested between the tundra bean goose and the pink‐footed goose (Ottenburghs, Megens, et al., 2016). Further studies are needed to elucidate the phylogenetic position of the pink‐footed goose and the possible admixture scenario.

### 4.5 Management implications

We did not find evidence to divide the Finnish bean goose population into smaller management units or subpopulations as there was no strong genetic structuring within Finland. Therefore, the flyway management units outlined in the International Single Species Action Plan (ISSAP; Marjakangas et al., 2015) seem to be justified based on our study (see Figure 2). The genetic diversity was found to be moderate and effective population size fairly large, thus the Finnish breeding taiga bean geese are not under immediate threat by genetic impoverishment. However, as the genetic diversity was lower compared to other bean goose subspecies and widespread goose species, further reductions in genetic diversity should be avoided to maintain the evolutionary potential of this subspecies, especially since inbreeding was detected in the population. Even though we found evidence of possible hybridization and introgression, naturally occurring hybridization does not pose a threat to populations as it is natural part of species evolution (Allendorf et al., 2001; Taylor & Larson, 2019). Thus, natural hybridization should not disqualify species from conservation programs and protection (vonHoldt et al., 2018).

Non‐invasive genetic sampling could be used for genetic monitoring of the taiga bean goose in the future to provide estimates of local population census sizes, survival, recruitment, and temporal variation in genetic diversity in order to ensure the genetic viability of the population (Schwartz et al., 2007). Besides microsatellites and mtDNA, SNP markers could be used in genetic monitoring, as SNPs do not require laborious calibration between different laboratories enabling flyway‐wide monitoring, and are well suited for non‐invasive samples as amplicon lengths are short and SNPs are less error prone than microsatellites (Carroll et al., 2018). A SNP panel has been already developed for subspecies identification for the greater white‐fronted goose (Wilson et al., 2019) and similarly a SNP panel could provide a feasible alternative for the genetic monitoring for the taiga bean geese.

## CONFLICT OF INTEREST

None declared.

## AUTHOR CONTRIBUTIONS

**Johanna Honka:** Conceptualization (lead); Formal analysis (lead); Investigation (lead); Methodology (lead); Visualization (lead); Writing – original draft (lead); Writing – review & editing (equal). **Serena Baini:** Investigation (equal); Writing – review & editing (equal). **Jeremy B. Searle:** Supervision (equal); Writing – review & editing (equal). **Laura Kvist:** Conceptualization (equal); Supervision (equal); Writing – review & editing (equal). **Jouni Aspi:** Conceptualization (equal); Supervision (lead); Writing – review & editing (equal).

## Supporting information

Supplementary Material

## Data Availability

The program code generated during the current study is available in the GitHub repository, https://github.com/karimerikanto/microsat_errcalc. The executable file is available on request from the corresponding author. The sequence data have been deposited in GenBank with accession numbers: MT023340–MT023341. The microsatellite genotypes are available for download in the Dryad repository: https://doi.org/10.5061/dryad.7wm37pvv6.
