# Supplementary material for: Genetic assessment reveals inbreeding, possible hybridization, and low levels of genetic structure in a declining goose population
Source: Ecol Evol. 2022 Jan 24;12(1):e8547. doi: 10.1002/ece3.8547 (PMC8796947; doi:10.1002/ece3.8547)
Supplement: Supplementary file 1 — Supplementary Material [file ECE3-12-e8547-s001.docx]

# Supplementary file

Genetic assessment reveals inbreeding, possible hybridization and low levels of genetic structure in a declining goose population

Johanna Honka^1*^, Serena Baini^2^, Jeremy B. Searle^3^, Laura Kvist^1^ and Jouni Aspi^1^

^1^ Ecology and Genetics Research Unit, University of Oulu, Oulu 90014, Finland

^2^Department of Biology, University of Rome “Tor Vergata", Rome 00133, Italy

^3^Department of Ecology and Evolutionary Biology, Cornell University, Ithaca, NY 14853, USA

*Corresponding author

email. [johanna.honka@oulu.fi](mailto:johanna.honka@oulu.fi)

**Supplementary table 1**. Summary statistics for 28 microsatellite loci genotyped in 488 taiga bean geese (*Anser fabalis fabalis*). Loci removed from the final analyses are marked with an asterisk. Also indicated are the multiplex sets to which the loci belong, the fluorescence label, primer concentration and whether the repeat being amplified is a dinucleotide, trinucleotide or tetranucleotide. Statistics: sample size (*n*), number of alleles (*A*), observed heterozygosity (*H*_O_), expected heterozygosity (*H*_E_), inbreeding coefficient (*F*_IS_), *P*-value from test of departure from Hardy-Weinberg equilibrium (*P*_HWE_), unbiased probability of identity (*P*_ID_), probability of identity of siblings (*P*_ID SIB_), null allele frequency (NULL), per-locus allele dropout rate (ADO) and per-locus false allele rate (FA). Statistically significant *P*-values after Bonferroni correction are showed in bold letters.

| Locus | **Multiplex set** | **Fluoro-label** | **Primer concent-ration (μM)** | **Repeat type** | **Allele size range (bp)** | ***n*** | ***A*** | ***H*_O_** | ***H*_E_** | ***F*_IS_** | ***P*_HWE_** | ***P*_ID_** | ***P*_ID_ _SIB_** | **NULL** | **ADO** | **FA** |
| --- | --- | --- | --- | --- | --- | --- | --- | --- | --- | --- | --- | --- | --- | --- | --- | --- |
| Afa05 | A | 6FAM | 0.25 | Tetra | 170-206 | 484 | 10 | 0.72 | 0.78 | 0.08 | **0.00** | 0.08 | 0.38 | 0.018 | 0.012 | 0.000 |
| Afa13 | A | NED | 0.25 | Tetra | 203-239 | 468 | 10 | 0.75 | 0.82 | 0.08 | **0.00** | 0.05 | 0.35 | 0.011 | 0.038 | 0.014 |
| Afa15 | A | 6FAM | 0.40 | Tetra | 219-255 | 476 | 9 | 0.68 | 0.77 | 0.12 | **0.00** | 0.08 | 0.39 | 0.021 | 0.048 | 0.024 |
| Afa25 | A | PET | 0.40 | Tetra | 220-260 | 456 | 10 | 0.67 | 0.79 | 0.15 | **0.00** | 0.07 | 0.38 | 0.021 | 0.062 | 0.009 |
| Afa30 | A | NED | 0.08 | Tetra | 108-188 | 486 | 17 | 0.82 | 0.85 | 0.04 | 0.42 | 0.04 | 0.33 | 0.012 | 0.037 | 0.009 |
| Afa33 | A | 6FAM | 0.06 | Tetra | 91-107 | 484 | 5 | 0.59 | 0.61 | 0.04 | 0.92 | 0.23 | 0.50 | 0.008 | 0.014 | 0.009 |
| Afa34 | A | VIC | 0.08 | Tetra | 209-233 | 466 | 8 | 0.64 | 0.74 | 0.14 | **0.00** | 0.10 | 0.41 | 0.021 | 0.062 | 0.002 |
| Afa02 | B | NED | 0.10 | Tetra | 190-234 | 478 | 10 | 0.73 | 0.79 | 0.08 | **0.00** | 0.07 | 0.37 | 0.000 | 0.039 | 0.008 |
| Afa17 | B | 6FAM | 0.40 | Tetra | 217-261 | 469 | 12 | 0.74 | 0.84 | 0.13 | **0.00** | 0.04 | 0.34 | 0.000 | 0.079 | 0.005 |
| Afa18* | B | PET | 0.10 | Tetra | 183-231 | 469 | 13 | 0.61 | 0.83 | 0.26 | **0.00** | 0.05 | 0.35 | 0.090 | 0.024 | 0.005 |
| Afa19 | B | 6FAM | 0.10 | Tetra | 167-187 | 484 | 6 | 0.54 | 0.57 | 0.05 | **0.00** | 0.27 | 0.53 | 0.013 | 0.021 | 0.002 |
| Afa35 | B | VIC | 0.06 | Tetra | 128-208 | 482 | 18 | 0.69 | 0.81 | 0.15 | **0.00** | 0.06 | 0.36 | 0.056 | 0.042 | 0.022 |
| Abra10 | C | 6FAM | 0.05 | Di | 144-170 | 485 | 14 | 0.72 | 0.79 | 0.09 | **0.00** | 0.07 | 0.37 | 0.000 | 0.028 | 0.005 |
| Abra14* | C | VIC | 0.05 | Di | 150-156 | 476 | 5 | 0.15 | 0.17 | 0.13 | **0.00** | 0.70 | 0.84 | 0.023 | 0.014 | 0.000 |
| Abra2 | C | VIC | 0.05 | Di | 91-107 | 481 | 10 | 0.58 | 0.67 | 0.13 | **0.00** | 0.15 | 0.46 | 0.056 | 0.017 | 0.020 |
| Abra23 | C | 6FAM | 0.30 | Di | 272-288 | 447 | 9 | 0.58 | 0.75 | 0.23 | **0.00** | 0.10 | 0.40 | 0.022 | 0.106 | 0.014 |
| Abra29* | C | NED | 0.10 | Di | 205-209 | 464 | 3 | 0.04 | 0.06 | 0.27 | **0.00** | 0.89 | 0.94 | 0.000 | 0.080 | 0.000 |
| Abra39 | C | NED | 0.05 | Di | 116-146 | 475 | 15 | 0.69 | 0.68 | -0.01 | 0.30 | 0.13 | 0.44 | 0.000 | 0.023 | 0.010 |
| Abra68 | C | 6FAM | 0.05 | Di | 120-130 | 487 | 5 | 0.37 | 0.42 | 0.12 | **0.00** | 0.38 | 0.64 | 0.012 | 0.033 | 0.005 |
| Abra9* | C | VIC | 0.05 | Di | 176-194 | 280 | 8 | 0.05 | 0.18 | 0.70 | **0.00** | 0.68 | 0.81 | 0.147 | 0.214 | 0.004 |
| Abra12 | D | NED | 0.20 | Di | 93-109 | 477 | 8 | 0.70 | 0.67 | -0.05 | 0.37 | 0.15 | 0.45 | 0.004 | 0.025 | 0.008 |
| Abra15* | D | FAM | 0.20 | Di | 181-201 | 448 | 9 | 0.40 | 0.75 | 0.47 | **0.00** | 0.10 | 0.40 | 0.193 | 0.063 | 0.017 |
| Abra19 | D | VIC | 0.10 | Tetra | 165-201 | 483 | 10 | 0.69 | 0.75 | 0.08 | 0.09 | 0.09 | 0.40 | 0.000 | 0.049 | 0.005 |
| Abra24 | D | 6FAM | 0.40 | Di | 289-295 | 410 | 4 | 0.22 | 0.32 | 0.30 | **0.00** | 0.50 | 0.72 | 0.045 | 0.150 | 0.003 |
| Abra30 | D | VIC | 0.10 | Tri | 107-122 | 484 | 4 | 0.32 | 0.33 | 0.02 | **0.00** | 0.49 | 0.71 | 0.018 | 0.048 | 0.007 |
| Abra43* | D | NED | 0.10 | Di | 120-138 | 483 | 8 | 0.45 | 0.64 | 0.30 | **0.00** | 0.17 | 0.47 | 0.102 | 0.078 | 0.015 |
| Abra5 | D | PET | 0.10 | Di | 119-139 | 464 | 10 | 0.73 | 0.77 | 0.04 | 0.02 | 0.09 | 0.39 | 0.014 | 0.033 | 0.008 |
| Abra7 | D | 6FAM | 0.20 | Di | 93-127 | 486 | 17 | 0.66 | 0.75 | 0.12 | **0.00** | 0.08 | 0.40 | 0.030 | 0.014 | 0.017 |
| All loci mean | |  |  |  |  | 465 | 9.5 | 0.55 | 0.64 | 0.15 | **0.00** |  |  | 0.034 | 0.041 | 0.009 |
| All loci cumulative | |  |  |  |  |  |  |  |  |  |  | 5E-25 | 4E-10 |  |  |  |

**Supplementary text 1**

The presence of historical bottlenecks was tested using the Wilcoxon sign-rank and the mode shift test in the program Bottleneck 1.2.02 (Piry, Luikart and Cornuet, 1999) with 1,000 iterations of the strict stepwise mutation model (SMM; Ohta and Kimura, 1973) or the two-phase model (TPM; Di Rienzo *et al*., 1994) with 95 % of stepwise mutations and variance of 12 as recommended for microsatellite markers by Piry, Luikart and Cornuet (1999). We also calculated the *M* ratio which is the mean ratio of the number of alleles to the allele size range (Garza and Williamson, 2001) using the program Arlequin 3.5.2.2. (Excoffier and Lischer, 2010).

No signs of recent population bottlenecks were detected based on Wilcoxon sign-rank test for excess of heterozygosity (all *P >* 0.99) in any of the geographical regions under SMM or TPM mutation models. The mode shift distribution showed a normal L-shaped distribution in all of the regions consistent with a lack of recent bottleneck. Rather, all geographic regions showed heterozygote deficiency based on Wilcoxon sign-rank test (all *P* < 0.005) indicating that geese in the regions are not under strict mutation-drift equilibrium. The mean *M* ratio (Garza-Williamson index) over all loci was 0.339 (standard deviation SD: 0.117) in the Finnish population. In each region separately the *M* ratio was 0.328 (SD: 0.112) for Western Finland, 0.348 (SD: 0.121) for Eastern Finland/Kainuu, 0.348 (SD: 0.112) for Northern Ostrobothnia/Southern Lapland and 0.336 (SD: 0.117) for Lapland. All *M* ratios in the Finnish population are below the threshold of 0.68 (Garza and Williamson, 2001) that indicates a population bottleneck.

**Supplementary Figure 1**. Newhybrids run using z options with simulated taiga bean goose (*Anser fabalis fabalis*), pink-footed goose (*A. brachyrhynchus*), F1, F2, backcross to taiga bean goose and backcross to pink-footed goose. Each simulated category included 100 individuals.


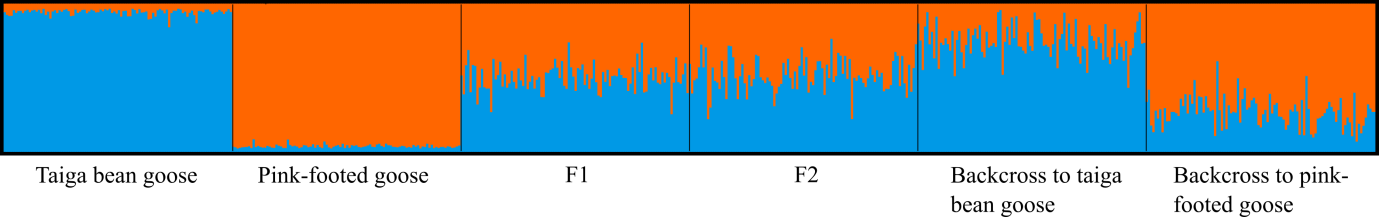


**Supplementary Figure 2**. Structure plot with *K* = 2 for simulated taiga bean goose, pink-footed goose, their F1 and F2 hybrids, backcrosses to taiga bean goose and backcrosses to pink-footed goose. Each vertical bar represents one individual with each simulated category including 100 individuals.

**Supplementary Figure 3**. Newhybrids run using z options with simulated taiga bean goose (*Anser fabalis fabalis*), tundra bean goose (*A. f. rossicus*), F1, F2, backcross to taiga bean goose and backcross to tundra bean goose. Each simulated category included 100 individuals.


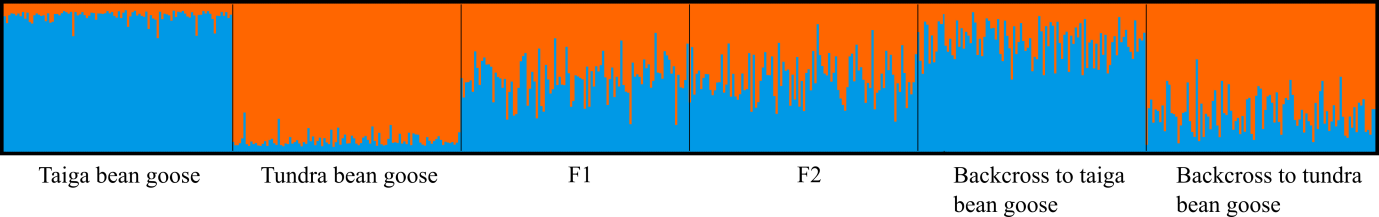


**Supplementary Figure 4.** Structure plot with *K* = 2 for simulated taiga and tundra bean goose, their F1 and F2 hybrids, backcrosses to taiga bean goose and backcrosses to tundra bean goose. Each vertical bar represents one individual with each simulated category including 100 individuals.


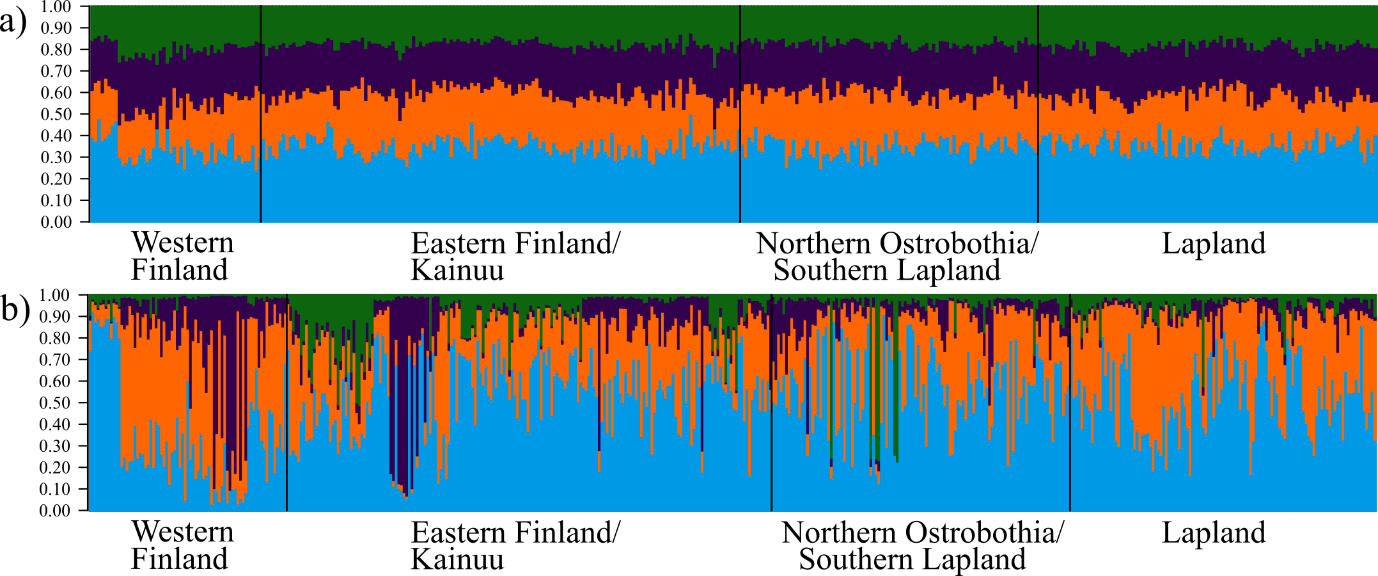


**Supplementary Figure 5.** Structure assignment plots for *K* = 4 using LOCPRIOR option for a) non-kin Finnish breeding taiga bean geese (*Anser fabalis fabalis*) (*n* = 376) and b) Finnish breeding taiga bean geese including closely related individuals (*n* = 488). Sampling locations within 16 km from each other were treated as one location. Each vertical bar represents one individual.

a)


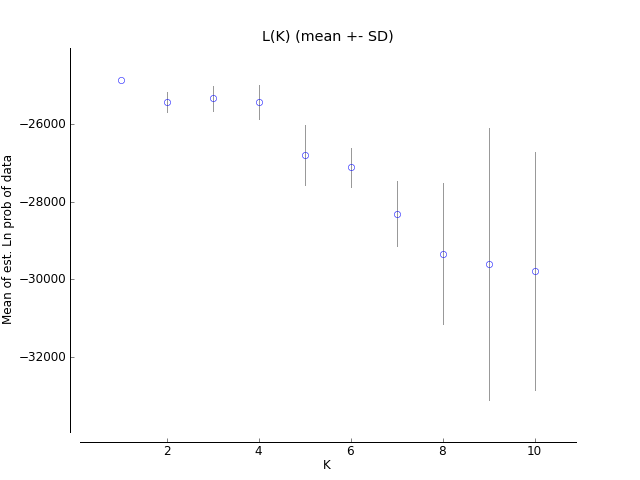


b)


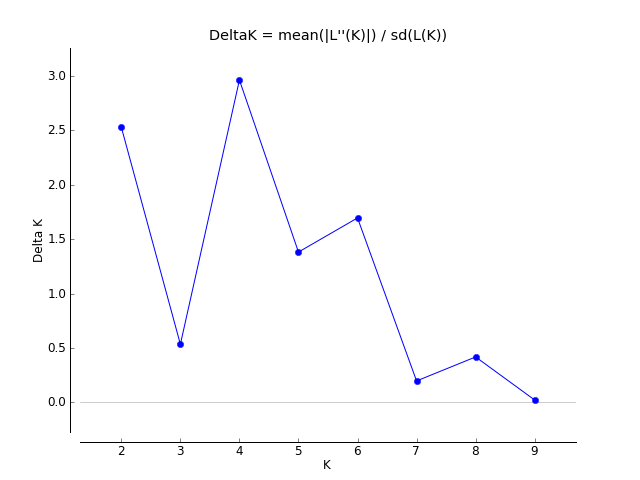


**Supplementary Figure 6**. a) Posterior likelihood graph L(K) from several Structure runs with different number of *K* and b) *ad hoc* statistics Δ*K* based on the method of Evanno *et al.* (2005) for non-kin Finnish breeding taiga bean geese (*Anser anser fabalis*) (*n* = 376).


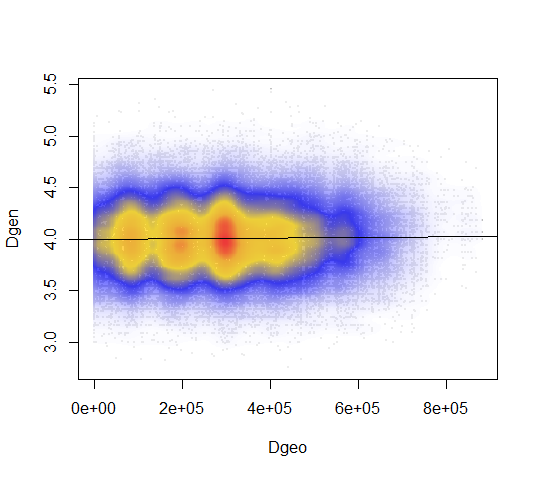


**Supplementary Figure 7**. Mantel test for isolation-by-distance using 376 unrelated bean goose breeding in Finland (*Anser fabalis fabalis*) with the genetic distance (Dgen) plotted against the geographic distance (Dgeo). The different colours represent different densities with warmer colours representing higher densities and cooler colours lower densities of samples. The black line shows the correlation between the matrices, *r =* 0.019.


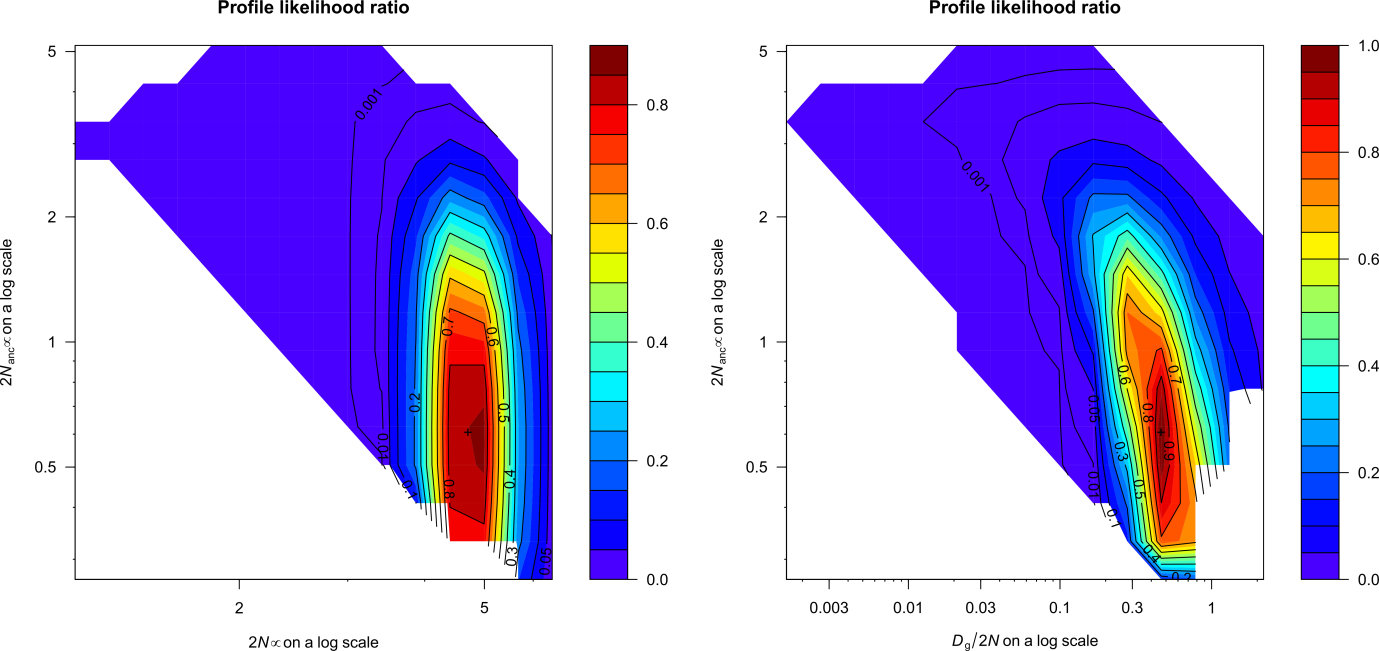


**Supplementary Figure 8**. Two-dimensional profile likelihood ratios for ancestral (2*N*_anc_) and current (2*N*) population sizes and timing of the demographic change (*D*g/2*N*) on a log scale for 488 bean geese breeding in Finland (*Anser fabalis fabalis*).

**References**

Di Rienzo, A., Peterson, A.C., Garza, J.C., Valdes, A.M., Slatkin, M. & Freimer, N.B. 1994, "Mutational processes of simple-sequence repeat loci in human populations", *Proceedings of the National Academy of Sciences of the United States of America,*vol. 91, no. 8, pp. 3166-3170.

Excoffier, L. & Lischer, H.E.L. 2010, "Arlequin suite ver 3.5: A new series of programs to perform population genetics analyses under Linux and Windows", *Molecular Ecology Resources,*vol. 10, no. 3, pp. 564-567.

Evanno, G., Regnaut, S. & Goudet, J. 2005, "Detecting the number of clusters of individuals using the software STRUCTURE: A simulation study", *Molecular Ecology,*vol. 14, no. 8, pp. 2611-2620.

Garza, J.C. & Williamson, E.G. 2001, "Detection of reduction in population size using data from microsatellite loci", *Molecular Ecology,*vol. 10, no. 2, pp. 305-318.

Ohta, T. & Kimura, M. 1973, "A model of mutation appropriate to estimate the number of electrophoretically detectable alleles in a finite population", *Genetical Research,* vol. 22, no. 2, pp. 201-204

Piry, S., Luikart, G. & Cornuet, J.-M. 1999, "BOTTLENECK: A computer program for detecting recent reductions in the effective population size using allele frequency data", *Journal of Heredity,*vol. 90, no. 4, pp. 502-503.
